# Investigation of the myopic outcomes of the newer intraocular lens power calculation formulas in Korean patients with long eyes

**DOI:** 10.1038/s41598-024-63334-y

**Published:** 2024-05-31

**Authors:** Jinchul Kim, Joonsung Park, Yoonjung Jo

**Affiliations:** Department of Ophthalmology, Miracle Eye Clinic, 115-gil, Teheran-ro, Gangnam-gu, Seoul, 06134 Korea

**Keywords:** Intraocular lens calculation formula accuracy, East Asian, Axial length modification, Myopia, Anterior chamber depth, Ethnic ocular biometric differences, Biophysics, Computational biology and bioinformatics, Diseases, Optics and photonics

## Abstract

This study investigated the underlying causes of the myopic outcomes of the optic-based newer formulas (Barrett Universal II, EVO 2.0, Kane, Hoffer-QST and PEARL-DGS) in long Korean eyes with Alcon TFNT intraocular lens (IOL) implantation. Postoperative data from 3100 randomly selected eyes of 3100 patients were analyzed to compare the reference back-calculated effective lens positions (ELPs) based on the Haigis formula using conventional axial length (AL) and Cooke-modified AL (CMAL) with the predicted ELP of each single- and triple-optimized Haigis formula applied to AL- and CMAL. Contrary to the AL-applied Haigis formula, the predicted ELP curve of the CMAL-applied, single-optimized Haigis formula, simulating the methods of the newer formulas, exhibited a significant upward deviation from the back-calculated ELP in long eyes. The relationship between the AL and anterior chamber depth in our long-eyed population differed from that in the base population of the PEARL-DGS formula. The myopic outcomes in long eyes appeared to stem from the substantial overestimation of the postoperative IOL position with AL modification, leading to the implantation of inappropriately higher-powered IOLs. This discrepancy may be attributed to the ethnic differences in ocular biometrics, particularly the relatively smaller anterior segment in East Asian patients with long AL.

## Introduction

The global prevalence of myopia is on the rise, particularly among the East Asian population^1–4^. Because moderate to high myopia is reportedly associated with cataracts, an increasing number of patients with myopia are expected to undergo cataract surgery in the future^3–5^, including those with a history of refractive laser procedures. While the refractive outcomes following cataract surgery have substantially improved, there is a consistent trend of obtaining hyperopic results in longer eyes^2–4,6–8^. Most optical biometers, except those utilizing the sum-of-segments axial length (AL), employ a group refractive index, regardless of their ability to measure each segment separately^9^, which reportedly may lead to shorter and longer AL measurements in short and long eyes, respectively^9–11^.

Various strategies have been proposed to address this issue, including empirically targeting myopia in postoperative refraction^3,4^ and utilizing the Wang–Koch (WK) adjustment methods for AL^6,11–14^. Recently, the Cooke-modified AL (CMAL) was developed to closely approximate the sum-of-segments AL^10^ and is considered less aggressive than the WK adjustment methods^5^.

Contrary to previous studies^2–4,6,7,15–17^, a report on the refractive outcomes of 3100 Asian eyes undergoing cataract surgery and multifocal Acrysof TFNT intraocular lens (IOL) (including toric versions; Alcon Laboratories, Fort Worth, TX, USA) implantation, revealed that the freely available newer IOL calculation formulas (Barrett Universal II, Emmetropia Verifying Optical (EVO) 2.0, Kane, Hoffer QST, and Prediction Enhanced by Artificial Intelligence and output Linearization—Debellemanière, Gatinel, and Saad [PEARL-DGS]) yielded significant myopic outcomes in long eyes (see Supplementary Fig. S1a online), which were not observed in the single- or triple-optimized, conventional AL applied Haigis formula (see Supplementary Fig. S1b online)^18^. In addition, the PEARL-DGS formula^19^ incorporated the CMAL for calculation and its prediction error demonstrated a high correlation (R^2^ > 0.85, R: Pearson correlation coefficient) with prediction errors of the Barrett, EVO 2.0, and Kane formulas. However, the R^2^ value decreased when the AL was replaced with the reversed CMAL (AL + 0.05467 × lens thickness [LT] − 1.23853)/0.95855)^18^, calculated using the conventional AL instead of the CMAL in its inner algorithm^19^ (see Supplementary Table S1 online). In comparison, when the CMAL was applied to the Haigis formula^20^ with single-constant optimization to simulate the functioning of the newer formulas (single constant, theoretical optics, and AL modification), it exhibited similar behavior and increased R^2^ values^18^.

This study aimed to investigate the underlying causes of the myopic shifts observed with optic-based newer formulas in long Korean eyes^18^. We hypothesized that these shifts might be attributed to differences in ocular biometric traits between the training populations of the newer optic-based formulas and our study population.

## Results

Tables 1, 2 presents the demographics and refractive outcomes of the entire study population and the subgroup with long-AL. Unlike the AL-applied Haigis formula (both single- and triple-optimized), all the newer formulas and the CMAL-applied, single-optimized Haigis formula exhibited statistically significant (*P* < 0.001, see Supplementary table S2 online) myopic mean numerical prediction error (ME). Consequently, this led to the refractive errors of the new formulas were significantly (*P* ≤ 0.01, see Supplementary table S3 online) higher than those of the Haigis (conventional AL-applied) formula when comparing the root mean square error (RMSE).

**Table 1:** Demographics and refractive results of the patient population. a: Demographics of the patient population.

Cases	Entire population (n = 3100)		Long eyes (AL > 26 mm, n = 133)	
		Range		Range
Right eye, n (%)	1586 (51.2)		70 (52.6)	
Female sex, n (%)	2287 (73.8)		84 (63.2)	
Age (years)	58.7 ± 5.7	38–87	55.1 ± 5.1	46–72
IOL power (D)	20.27 ± 3.40	6–31	11.47 ± 2.5	6–17
Axial length (mm)	23.68 ± 1.15	20.57–29.73	26.77 ± 0.70	26.02–29.73
Mean conventional keratometry (D)	44.21 ± 1.40	39.63–50.50	43.53 ± 1.36	39.62–47.30
Mean total keratometry (D)	44.18 ± 1.40	39.41–50.28	43.52 ± 1.38	39.40–47.20
Anterior chamber depth (mm)	3.14 ± 0.35	1.89–4.30	3.51 ± 0.27	2.65–4.09
Lens thickness (mm)	4.47 ± 0.31	3.37–5.66	4.31 ± 0.26	3.67–4.87
Central corneal thickness (µm)	541.35 ± 32.23	434–685	543.02 ± 34.36	454–662
Corneal diameter (mm)	11.81 ± 0.38	10.5–13.7	11.94 ± 0.39	10.79–12.87
Postoperative SE (D)	-0.086 ± 0.33	-1.25–1.0	-0.275 ± 0.34	-1.25–0.5

*IOL* intraocular lens, SE spherical equivalent.

**Table 2:** Demographics and refractive results of the patient population. b Refractive results of the patient population.

Formula	Whole group (n = 3100)		Long eyes (> 26 mm, n = 133)		
	ME	SD	ME	SD	RMSE
PEARL-DGS	− 2.0E− 06	0.3175	− 0.2933	0.3410	0.4471
HTCL	− 0.0015	0.3133	− 0.0585	0.3344	0.3383
Kane	0.0001	0.3168	− 0.2245	0.3347	0.4019
EVO 2.0	− 1.2E−05	0.3171	− 0.2707	0.3409	0.4343
HTAL	− 0.0019	0.3221	− 0.0273	0.3385	0.3383
HSAL	0.0006	0.3226	− 0.0062	0.3400	0.3388
HSCL	2.4E−05	0.3260	− 0.2323	0.3433	0.4135
Barrett	0.0002	0.3282	− 0.2420	0.3377	0.4144
Hoffer QST	− 0.0001	0.3358	− 0.2702	0.3513	0.4421
Holladay 1	− 3.4E−06	0.3505	0.0987	0.3909	0.4018
Hoffer Q	− 5.8E−06	0.3534	0.1150	0.3913	0.4065
SRK/T	− 0.0004	0.3902	− 0.1222	0.3923	0.4095

*AL* axial length, *CMAL* Cooke-modified axial length, *HSAL* Haigis formula single-optimized and AL-applied, *HSCL* Haigis formula single-optimized and CMAL-applied, *HTAL* Haigis formula triple-optimized and AL-applied, *HTCL* Haigis formula triple-optimized and CMAL-applied, *ME* mean numerical prediction error, *SD* standard deviation, *EVO* Emmetropia Verifying Optical formula, Hoffer QST Hoffer Q/Savini/Taroni formula, *PEARL–DGS* Prediction Enhanced by Artificial Intelligence and output Linearization–Debellemanière Gatinel and Saad, *RMSE* root mean square numerical error, *SD* standard deviation.

There were discrepancies in the back-calculated reference effective lens position (ELP) between the AL- and CMAL-applied Haigis formulas (see Supplementary Fig. S2 online). Specifically, our findings indicated that the reference ELP of the CMAL version was higher and lower at an AL < 24 mm and > 24 mm, respectively.

**Figure 1:**
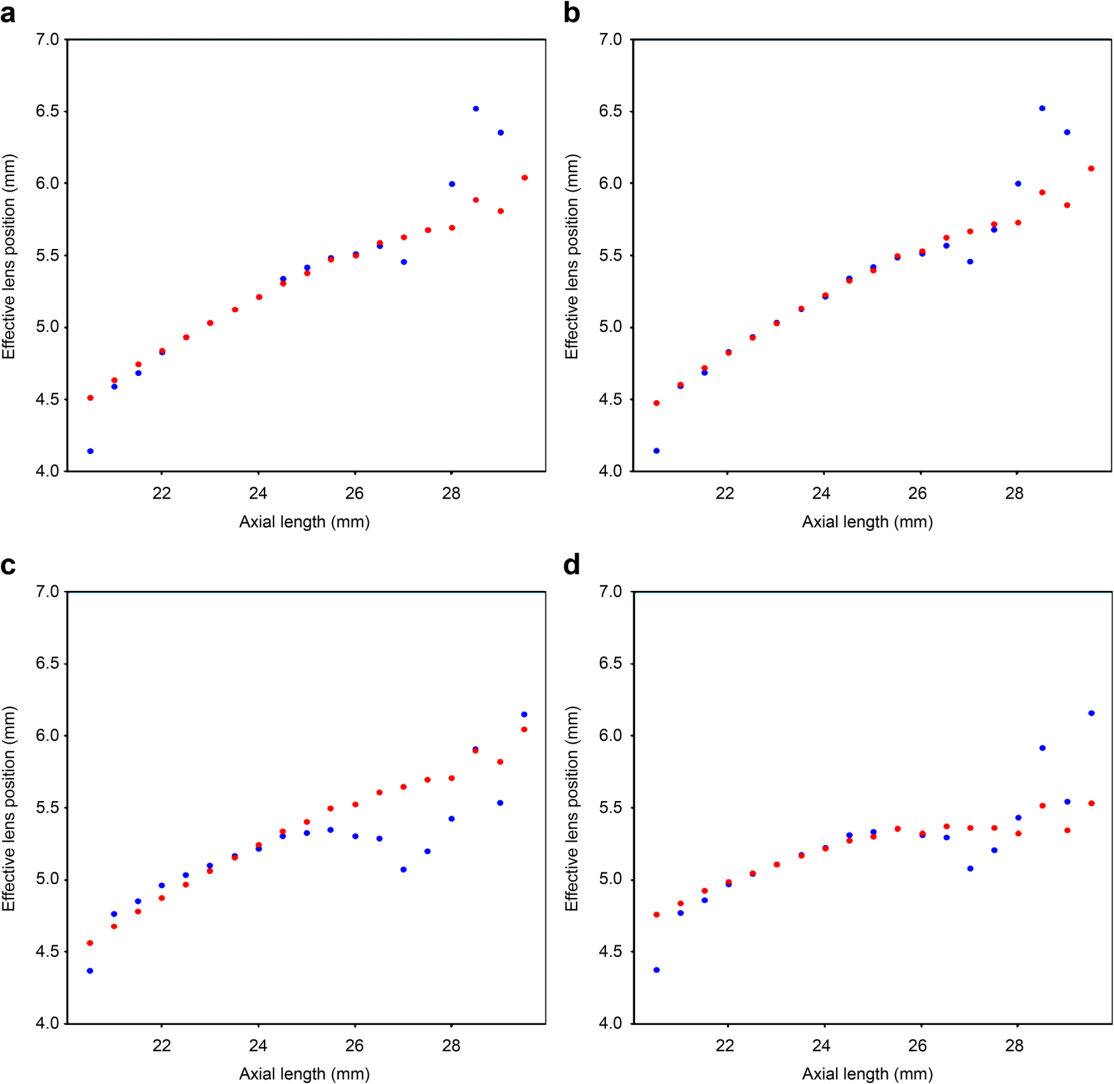
Comparison between the back-calculated ELP versus the predicted ELP of the Haigis formula, with single and triple optimization. Mean ELP values are plotted against conventional AL values rounded to 0.5 mm. (**a**) Haigis formula with the conventional AL application, single-optimized (a0/a1/a2 constants: 1.523/0.4/0.1), (**b**) Haigis formula with the conventional AL application, triple-optimized (a0/a1/a2 constants: 1.304/0.442/0.104), (**c**) Haigis formula with the Cooke-modified axial length application, single-optimized (a0/a1/a2 constants: 1.556/0.4/0.1), (**d**) Haigis formula with the Cooke-modified axial length application, triple-optimized (a0/a1/a2 constants: 3.526/0.523/0).

While no remarkable difference was observed in the predicted ELP between the AL and CMAL versions with single optimization, the difference in the reference back-calculated ELP between them was more pronounced when the AL was > 24.5 mm (Fig. 1). The near-linear relationship between the AL and the reference ELP was maintained in the AL version (Fig. 1a,b) but showed deviations in the CMAL version when the AL was > 24.5 mm (Fig. 1c,d).

**Figure 2:**
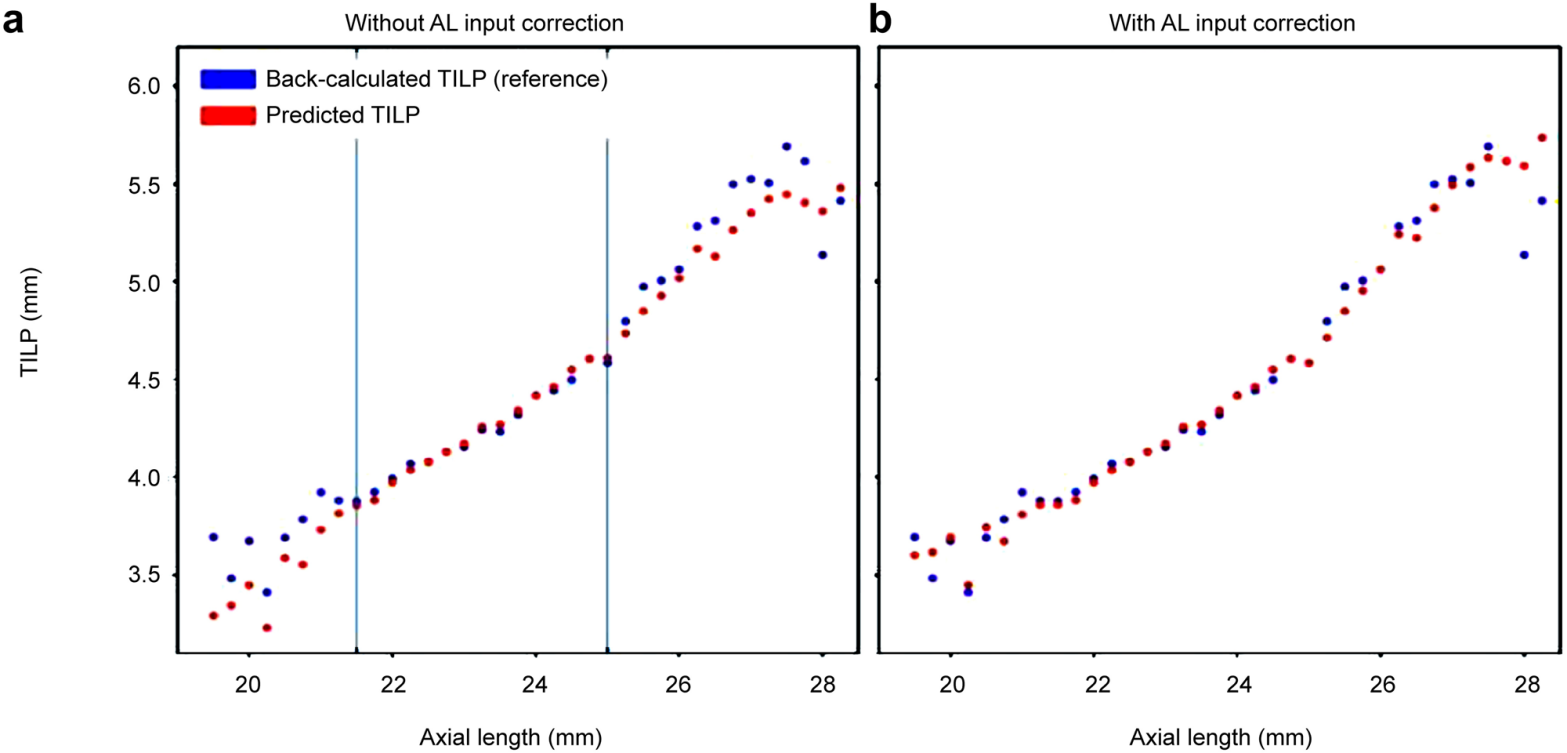
Comparison between the back-calculated theoretical internal lens position (TILP) versus predicted TILP in the PEARL-DGS formula training population (figure derived from reference 19, the units of measurement were revised for consistency.) Mean TILP values are plotted against conventional AL values rounded to 0.25 mm. (**a**) In the long eyes, the back-calculated TILP is higher than the predicted TILP, which is contrary to our data (see Fig. 1c), (**b**) After AL input correction, in the long eyes, the predicted TILP is pushed upwards to fit the back-calculated TILP. This refinement would lead to more myopic deviation in our population’s long eyes. AL = axial length; TILP = theoretical internal lens position.

In the training population of the PEARL-DGS formula, the linear relationship was disrupted when the AL was > 25 mm. The reference theoretical internal lens position (TILP), equivalent to the reference ELP in thin lens formulas, was higher than the uncorrected predicted TILP (Fig. 2).

In the triple-optimized CMAL-applied Haigis formula, the predicted ELP demonstrated a noticeable adjustment to the reference ELP by flattening the upward slope when the AL was > 25.5 mm (Fig. 1d).

**Figure 3:**
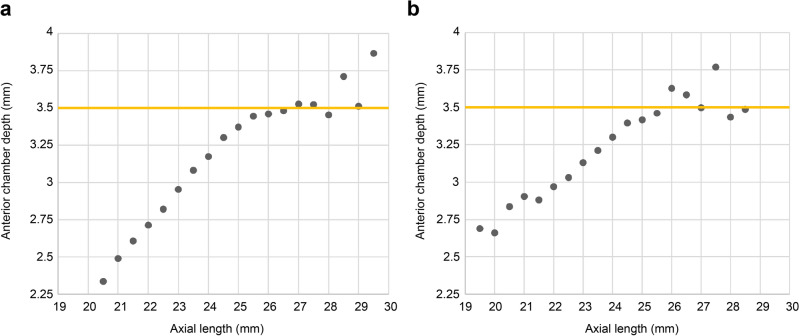
The relationship between the AL and the anterior chamber depth (ACD). Mean ACD values are plotted against conventional AL values rounded to 0.5 mm. (**a**) A: Relationship in our population (n = 3100), (**b)** B: Relationship in the PEARL-DGS formula training population (n = 4242, data from the formula author, image has been modified for easier comparison by us and confirmed for usage by the formula author).

Figure 3 illustrates the AL-anterior chamber depth (ACD; measured from the corneal epithelium to the anterior lens surface) relationship in our study population and the base population of the PEARL-DGS formula. The upward slope flattened when AL > 25.5 mm, and the ACD deepening ceased at approximately 3.5 mm in our study population. While the linear upward slope was disrupted in the PEARL-DGS formula population, an increase in ACD above 3.5 mm was still evident when AL > 25.5 mm.

In the subgroup analysis of ACD, all formulas exhibited similar slopes, showing increased myopia in the short ACD group (see Supplementary Fig. S3 online). The newer formulas and single-optimized CMAL-applied Haigis formula showed markedly higher myopic deviations across all ACD ranges. The slope was less evident in the shorter AL range (see Supplementary Fig. S4 online).

When using conventional AL application, the SRK/T formula showed myopic deviations in AL > 26 mm, whereas the Holladay 1 formula exhibited myopic deviations in eyes with AL between 25 and 26 mm. The Hoffer Q formula demonstrated relatively smaller hyperopia in long eyes (see Supplementary Fig. S5a online). With the CMAL application, all older formulas displayed a clockwise rotation, deviating towards myopia in long eyes (see Supplementary Fig. S5b online).

## Discussion

Besides the sum-of-segments AL-based biometer, optical biometers rely on a group refractive index for AL measurement^9,21^. However, they reportedly underestimate and overestimate the AL in short and long eyes, yielding myopic and hyperopic outcomes, respectively^9,11^. Several solutions, including AL modification methods^11^, were proposed to address this issue. Among them, the CMAL^10^, a more recent and sophisticated approach designed to simulate the sum-of-segments AL, reportedly provides better accuracy in non-standard eyes^5^. Contrary to previous studies^2–4,6,7,15–17^, a report on the refractive outcomes of 3100 Korean eyes that underwent cataract surgery and multifocal Acrysof TFNT IOL implantation showed that the freely available newer IOL calculation formulas yielded significant myopic results in long eyes (*P* < 0.001, see Supplementary Table S2 and Supplementary Fig. S1 online), which led to a deterioration in refractive outcomes (see Supplementary Table S3 online)^18^. Thus, our study aimed to further explore these conflicting results observed in previous studies regarding the myopic shifts associated with optic-based newer formulas in Korean patients with long eyes.

The PEARL-DGS formula incorporates the CMAL for calculations^19^. A previous report demonstrated that, except for the Hoffer QST formula, the correlation between the PEARL-DGS and other formulas decreased when reversed CMALs, which translated into conventional ALs in the PEARL-DGS formula online calculator, were entered into the PEARL-DGS formula. However, the correlation between the single-optimized Haigis and newer formulas increased when CMAL was applied to the Haigis formula (see Supplementary Table S1 online)^18^. Both results support our speculation that the other newer formulas also employ similar AL modifications, which aligns with the findings of a previous report^21^. Based on these findings, we explored the underlying causes for myopic shifts by investigating the PEARL-DGS and Haigis formulas.

The accuracy of the formulas with an optical backbone is primarily determined by how closely they can approximate the ELP necessary for the actual postoperative refraction^22,23^. In the implantation of thinner IOLs in long eyes^24,25^, the back-calculated reference ELP of the thin lens formulas (including the Haigis formula) would presumably not significantly differ from the back-calculated TILP of the PEARL-DGS formula^19^ or any equivalent concept regarding IOL position in other formulas. While the predicted and back-calculated ELP approximated the necessary ELP appropriately when the AL was < 28 mm in the conventional AL versions (Fig. 1a, b), an evident deviation was observed between them when the AL was > 24.5 mm in the single-optimized CMAL version (Fig. 1c). Thus, we considered that the shortened CMAL in the long AL range might have reduced the required ELP for achieving emmetropia (see Supplementary Fig. S2 online). Consequently, the near-linear relationship between the AL and reference ELP, which was maintained in the AL version, was disrupted in the CMAL version when the AL was > 24.5 mm.

During the analysis of eyes with an AL > 26 mm, we found a mean TK value of 43.52 D and a mean IOL power of 11.47 D (Tables 1, 2). The mean ELP prediction error, calculated as the difference between the mean reference ELP (5.28 mm) and the mean predicted ELP (5.63 mm) using the single-optimized CMAL-applied Haigis formula, was − 0.35 mm. This resulted in a mean refractive error of -0.24 D, which closely corresponded to the theoretical simulation by Gatinel et al.^24^. From Fig. 3 in that study, we could estimate that an ELP prediction error of − 0.35 mm in a pseudophakic eye model with 43 D corneal power and 11.5 D IOL power would lead to an approximate refractive error of − 0.25 D (see Supplementary Fig. S6 online) ^24^.

**Figure 4:**
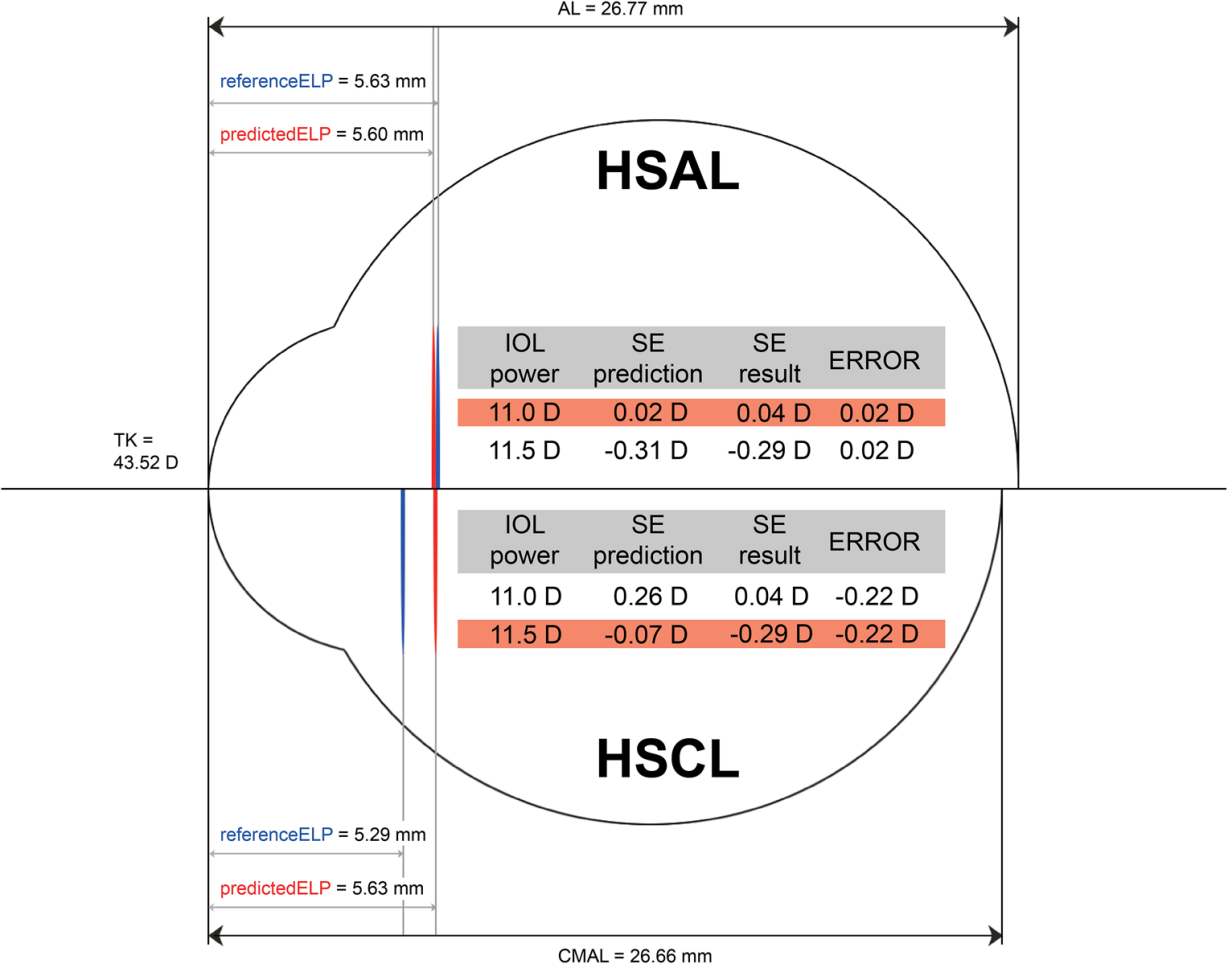
An eye model based on the mean values of the long-eye subgroup. Upper half: Eye model applying Haigis formula, single-optimized and AL-applied (HSAL). Lower half: Eye model applying Haigis formula, single-optimized and CMAL-applied (HSCL). AL = axial length; CMAL = Cooke-modified axial length; ELP = Effective lens position; HSAL = Haigis formula, single-optimized and AL-applied; HSCL = Haigis formula, single-optimized and CMAL-applied; IOL = Intraocular lens; SE = Spherical equivalent; TK = Total keratometry.

Figure 4, drawn from the mean values in our subgroup with long-eye (Tables 1, 2), illustrates the differences in ELPs and the ensuing impacts on the postoperative refractions between the single-optimized, AL-applied, and single-optimized, CMAL-applied Haigis formulas. The CMAL version showed an overestimation of the ELP, leading to a recommendation of an 11.5 D lens, while the AL version suggested an 11.0 D lens. The use of a higher-powered IOL led to myopic outcomes. Similar trends were observed in the newer formulas included in this study (see Supplementary Fig. S7 online (from the European Society of Cataract and Refractive Surgeons online calculator: https://iolcalculator.escrs.org/). All these formulas, using their respective optimized constants, favor the implantation of an 11.5 D lens over an 11.0 D lens.

With single optimization, the near-linear ELP prediction algorithm of the Haigis formula cannot adequately adapt to non-linear relationships. As the single optimization typically modifies the a0 constant while keeping the a1 and a2 constants unchanged, it merely shifs the intercept of the predicted ELP curve upward or downward^23^ without altering its curvature. The presence of a downward bending in an AL > 24.5 mm, as observed in the AL-reference ELP relationship graph in the CMAL version, suggests that while it may be a reasonable assumption that the predicted ELP is positively influenced by AL change (a2 > 0), it cannot adapt adequately to the reference ELP in long eyes of our population.

The IOL position prediction algorithms are fundamentally designed to fit their training populations’ back-calculated IOL position. As a result, they inherently mirror the ocular biometric traits specific to those training populations. In the newer formulas that utilize single constants, regardless of the number of variables additionally incorporated, the coefficients or other mechanisms that dictate the weight of their contribution to the IOL position prediction remain pre-determined^19^. In view of these facts, when the ocular biometric characteristics of the training population from which the novel formulas were developed vary from those of the testing population, similar behavior to the single-optimized CMAL-applied Haigis formula in our data could be encountered.

The relationship between the reference back-calculated TILP and the predicted TILP within the training set of the PEARL-DGS formula (Fig. 2a) differed from that between the reference ELP and the predicted ELP within our population using the CMAL-applied, single-optimized Haigis formula (Fig. 1c). In the training population of the PEARL-DGS formula, contrary to our results, the reference TILP was higher than the uncorrected predicted TILP. For compensation, the PEARL-DGS formula integrated an additional AL input correction algorithm to raise the predicted TILP toward the reference TILP (Fig. 2b)^19^. This algorithm, designed for further polishing, would have induced even higher myopic shifts than without it. This can be regarded as an overfitting problem in machine learning^26^; an approach to better fit the model to the training dataset results in underperformance in a different dataset.

Contrarily, when the CMAL-applied Haigis formula underwent triple optimization, the predicted ELP demonstrated a noticeable adaptation to the reference ELP by altering the upward slope down when the AL was > 25.5 mm (Fig. 1d). The triple optimization of the Haigis formula can be considered a complete retraining of the formula^19^, enabling the results to be consistent, irrespective of the AL used^21,27^. In line with previous reports^21,27^, using modified ALs resulted in an increase in the a1 constant and a decrease in the a2 constant of the Haigis formula compared to using conventional AL (Table 3). However, in our study population, the a2 constant decreased to 0. This nullification of the a2 constant, which severs the influence of the AL change on the ELP prediction, enabled this adaptation. Consequently, the overall SD of the Haigis formula decreased remarkably (Table 1)^18^.

**Table 3:** Triple-optimized a1 and a2 constants of the Haigis formula in two axial length groups from different studies.

Study	Conventional AL		Modified AL	
	a1	a2	a1	a2
Our study (N = 3,100)	0.442	0.104	0.523	0
Cooke and Cooke ^21^ (N = 1,442)	0.304	0.221	0.4	0.118
Shammas et al. ^27^ (N = 595)	0.204	0.234	0.323	0.149
Default values ^20^ (Used in the HSCL to simulate the newer formulas)	0.4	0.1	0.4	0.1

*AL* axial length, *HSCL* Haigis formula, single-optimized and CMAL-applied.

With the ACD becoming the primary factor governing ELP prediction, we investigated the ACD change based on AL, anticipating differences between the two population groups. Numerous studies have indicated that East Asian populations tend to have smaller anterior segments, including shallower ACDs than Caucasians^28,29^.

Figure 3 shows the AL-ACD relationship in our population and the training population of the PEARL-DGS formula. The resemblances between the ACD and CMAL-applied Haigis formula’s back-calculated ELP changes over the AL (Fig. 1c,d) were evident. The upward slope flattened when the AL was > 25.5 mm, and the ACD deepening ceased around 3.5 mm in our population (Fig. 3a), making the mean ACD of long eyes 3.51 mm (Tables 1, 2). Contrarily, although the linear upward slope was also disrupted in the PEARL-DGS formula population (courtesy of the author), a further increase of over 3.5 mm in the ACD was still evident when the AL was > 25.5 mm (Fig. 3b). When considering a near-normal distribution and setting a cutoff for a long AL at 26 mm, as widely accepted^1,4–7,16,18,19,21,30,31^, the mean ACD for ALs > 26 mm will be predominantly affected by the ACDs of eyes with ALs near the 26 mm mark, where the densest cluster of data points is located. Therefore, it could result in the TILP prediction algorithm still being positively influenced by AL changes in AL > 26 mm.

The myopic tendencies of the newer formulas in the long AL range decreased in the long-ACD subgroup (see Supplemental Figures S3and S4 online). This finding suggests that if the long-eye group had longer ACDs, there could be less myopic deviations, supporting our hypothesis that the IOL position prediction algorithms of the newer formulas may have been trained using populations that include eyes with longer ACDs than our study population in the long AL range.

This biometric difference could also offer an additional explanation. It has been known that the optical biometers’ overestimation of AL in long eyes can induce hyperopia in ultrasound-derived older formulas^10–12,21,27,32^. However, in our study population, the more anterior positioning of the IOL, attributable to the plateauing of ACD deepening (Fig. 3a), similar to the effect of the AL modification, may have mitigated the hyperopic tendency. This could result in a smaller hyperopia (Hoffer Q and Holladay formulas) or even myopia (SRK/T formula, see Supplementary Fig. S5a online), resembling the older formulas using sum-of-segments AL in Cooke and Cooke's study^21^. In Table 3, a notable similarity is also found among the Haigis formula’s constants (a1 and a2) across three different sets: the default values, the values from our study population using conventional AL, and those in other studies^21,27^, employing modified AL rather than conventional AL. This suggests that our study population with conventional AL may exhibit a behavior comparable to that of the Caucasian population with AL modification. Also, this similarity justifies our experimental simulation of the behavior of the newer formulas using a single-optimized, CMAL-applied Haigis formula. Furthermore, these findings may provide insight into why the reversed CMAL (AL + 0.05467 × lens thickness [LT] − 1.23853)/0.95855), when applied to the PEARL-DGS formula and calculating with the conventional AL in place of the CMAL in its inner algorithm^19^, led to increased stability for AL changes (see Supplementary Fig. S1d online) and a significantly lower overall SD (0.302 versus 0.318, *P* < 0.001) in a previous study^18^. Consequently, when the AL modification algorithms were applied to our population, as in the newer formulas (see Supplementary Fig. S1a online), single-optimized CMAL-applied Haigis formula (see Supplementary Fig. S1c online), or the CMAL-applied older formulas (see Supplementary Fig. S5b online), they could potentially overcompensate and induce more aggressive myopic deviations in long eyes.

**Figure 5:**
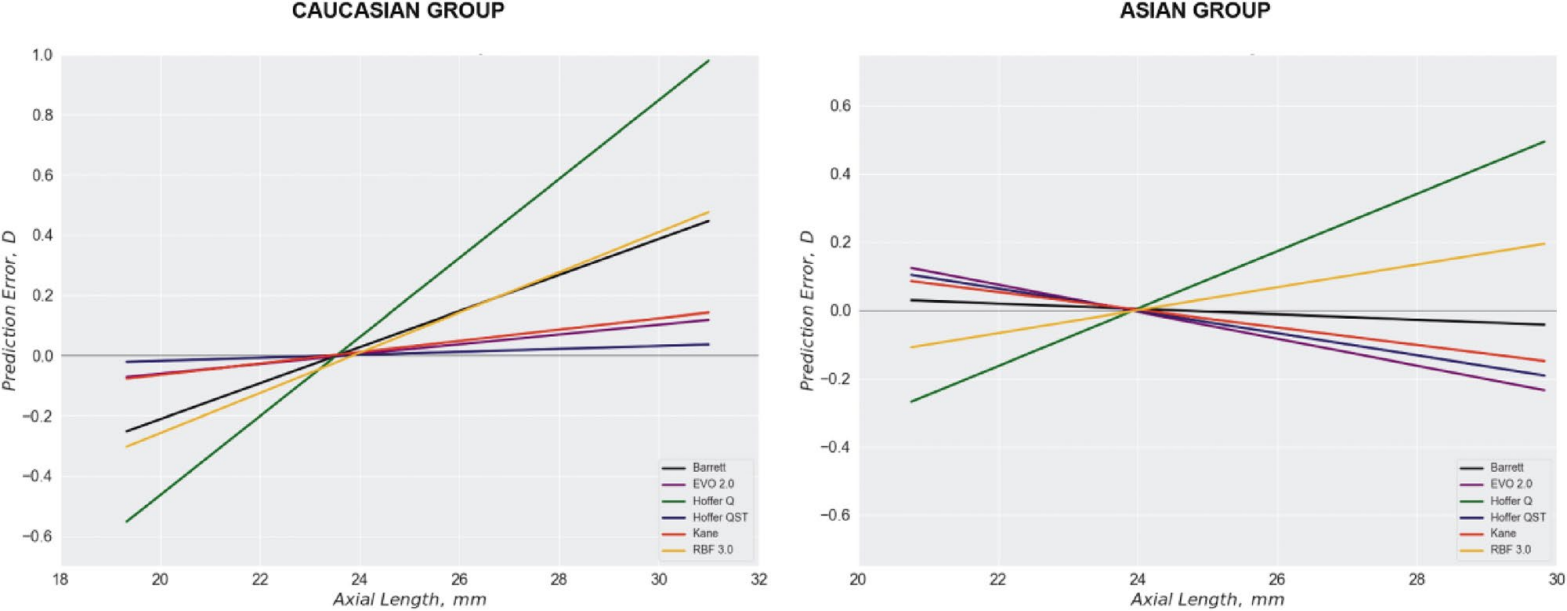
Mean prediction error changes of each formula according to AL (figure derived from Reference 33, and modified to enhance visual clarity). EVO: Emmetropia Verifying Optical formula; Hoffer QST: Hoffer Q/Savini/Taroni formula; RBF: Hill Radial Basis Function formula.

Comparable findings were also observed in a recent study by Taroni et al.^33^, which included results from both Japanese and Caucasian groups. In that study, all formulas displayed a clockwise rotation in the Japanese group; more myopic in long eyes and more hyperopic in short eyes compared to the Caucasian group, resulting in a reversal of the slope sign from positive to negative in the Barrett, EVO 2.0, Hoffer QST, and Kane formulas (Fig. 5). A similar clockwise rotation with modified AL implementation, compared to conventional AL was also evident in our results (see Supplementary Figs. S1c, S1d, and S5 online), as well as in previous studies^21,32^.

**Figure 6:**
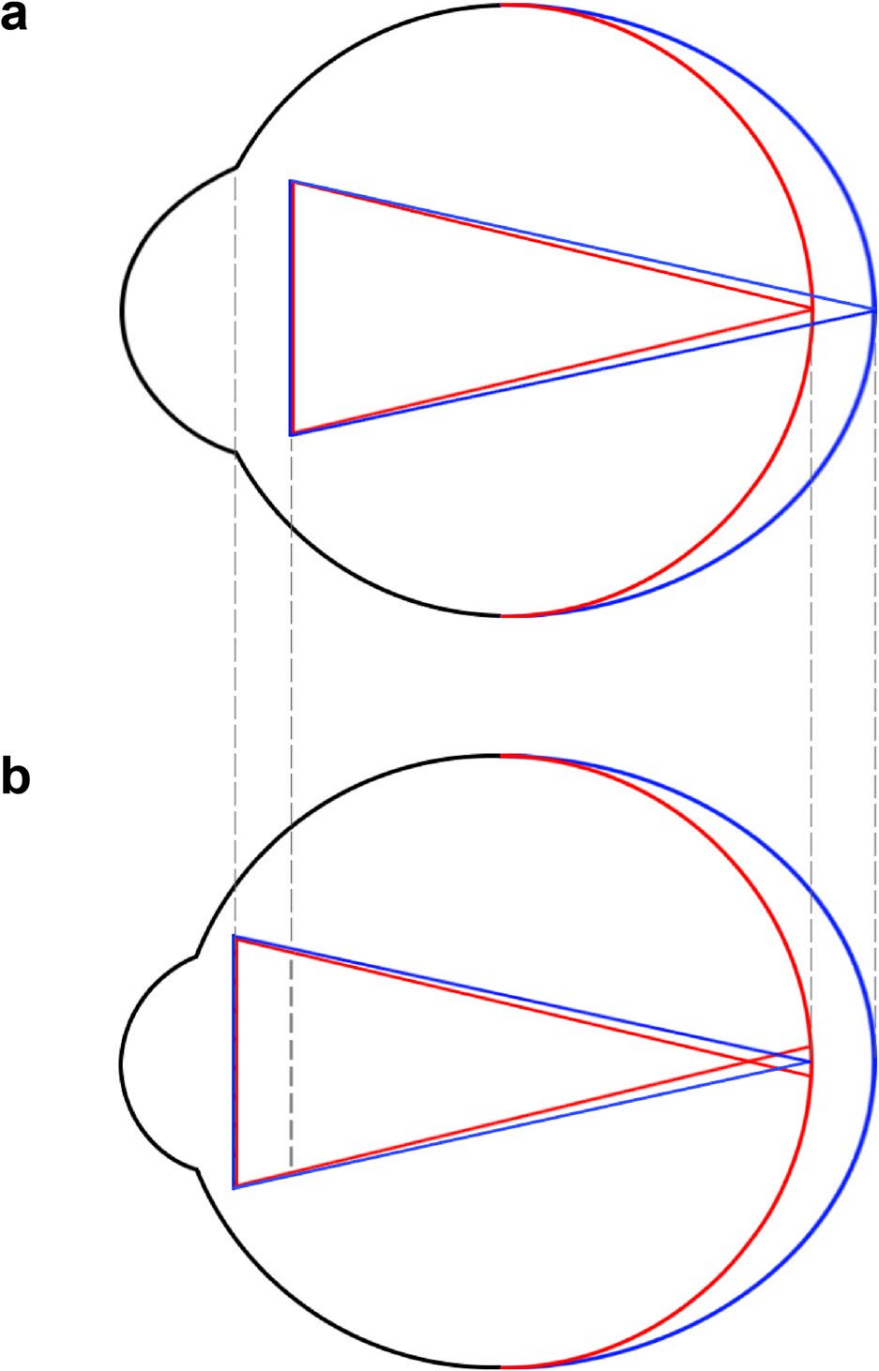
Ethnic Variations in Ocular Biometric Characteristics and their Impact on Refractive Outcomes in Long Axial Length Range. (**a**) Long Eyes in Caucasian patients with bigger anterior segments, (**b**) Long Eyes in East Asian patients with smaller anterior segments. Blue-colored hemicircle: Eye model with conventional AL. Red-colored hemicircle: Eye model with modified AL, approximating true AL. Blue-colored triangle: Lower-powered IOL and its longer focal length.Red-colored triangle: Higher-powered IOL and its shorter focal length. AL = axial length; IOL = Intraocular lens.

These results suggest that, as depicted in Fig. 6, efforts to address hyperopia in the long eyes of the Caucasian population, such as AL modification algorithms, may result in myopic overcompensation in East Asian population due to differences in ocular biometric characteristics.

To the best of our knowledge, this study represents the first attempt to describe the mechanisms of how ethnic differences in ocular geometry can specifically influence the outcomes of IOL power calculation formulas. While there have been suggestions that variations in ocular characteristics among different ethnic groups can impact refractive outcomes in cataract surgery^28,33^, detailed descriptions of the mechanisms by which these variations influence the outcomes have not been reported. By reasonably employing available methods in a predominantly independent manner, this study proposes a plausible hypothesis for the underlying causes in the myopic outcomes of newer IOL calculation formulas in long Korean eyes. The newer formulas are mostly proprietary and unpublished; therefore, they have commonly been considered difficult for scientific discussion^21,25,34^.

East Asia alone comprises more than half of the world's myopic population^2^, with a growing trend^1–4^. In addition, the myopic tendencies observed in the present study were found to be not only restricted to ALs > 26 mm, on which this study focused, but also appeared in the medium-long AL range with lower magnitude (see Supplementary Fig. S1 online). Therefore, this study may stimulate a significant consideration among cataract surgeons, particularly in those who are implanting the same IOLs in East Asian patients.

This study has several limitations. Our analysis of the Caucasian population relied on the courtesy of other authors and inferences drawn from existing literature. Having direct access to data from both ethnicities, as available in another study^33^, would have permitted a more detailed analysis, including statistical comparisons. Additionally, the majority of the findings were based on eyes with an AL range between 26 and 28 mm. Due to the power range of the studied IOL (6.0 D or higher), only six eyes had an AL above this range, which is insufficient to derive meaningful conclusions. Thus, it is speculated that for eyes with longer ALs, which would typically require lower-powered IOLs, the ELP prediction error might have a smaller impact on refractive outcomes^24,35^. Therefore, the ethnic divergence of ocular biometric characteristics and its impact on refractive outcomes discussed in this study may not be generalizable to eyes with longer ALs. Future studies, including a sufficient number of eyes with longer ALs, may address this issue further.

Our results contradict previous acumen; hyperopic surprise following cataract surgery should be cautioned in long eyes^2–4,6–8^, and newer formulas could be more accurate throughout the AL range^6,15,16^. Whether our findings can be reproduced in the East Asian population needs validation by future research. A recent article about the same IOLs from a similar clinical setting (including a Korean population with the same biometer) demonstrated comparable outcomes of the Barrett (− 0.22 D), SRK/T (− 0.06 D), and Hoffer Q (0.07 D) formulas in an AL > 26 mm (98 eyes) when their MEs in the total ALs (2018 eyes) was adjusted to 0^30^, which aligns with our results (Table 1, 2).

In conclusion, the recently reported significant myopic outcomes of the newer formulas in long Korean eyes with TFNT IOL implantation^18^ appear to originate from the substantial overestimation of the postoperative IOL position, which becomes more pronounced with AL modification. This consequently induced the implantation of higher-powered IOLs than necessary for achieving emmetropia. This can be attributed to the ethnic ocular biometric difference, particularly the relatively smaller anterior segment in East Asian patients with long AL. Considering the potential clinical consequences when combined with conventional myopic targeting, cataract surgeons performing multifocal IOL implantation in myopic East Asian patients should be aware of this phenomenon. Additionally, when developing or evaluating formulas, researchers should take this phenomenon into consideration.

## Methods

This study conformed to the tenets of the Declaration of Helsinki and was approved by the Korean Public Institutional Review Board. Owing to anonymized data extraction and analysis, the requirement for informed consent was waived by the Korean Public Institutional Review Board.

The dataset of this study has been previously described^18^. Data from 3100 randomly selected eyes of 3100 patients who underwent cataract surgery with trifocal Acrysof TFNT IOL (including toric versions; Alcon Laboratories) implantation from January 2020 to April 2022 at an eye clinic in Seoul, Korea, were retrospectively reviewed. Biometric measurements were taken for all patients preoperatively with the IOLMaster 700 (software versions 1.88 to 1.90, Carl Zeiss Meditec, Jena, Germany). The total keratometry (TK) values were used by default.

The selection of IOL power in practice was based on the biometer printout, which provided results of four formulas using default constants: SRK/T (A-constant: 119.1), Hoffer Q (pACD: 5.61), Haigis (a0: 1.390, a1: 0.4, a2: 0.1), and Barrett (LF: 1.94, equivalent to A-constant 119.1 on the online calculator). In situations where there were discrepancies among the recommendations,the recommendation of the Barrett formula was primarily followed,

For 26 patients who underwent IOL exchange following cataract surgery, the predicted refraction of the finally implanted IOL power with the biometric data before cataract surgery was used for calculation. The final postoperative refraction was collected at least 3 months after the cataract surgery or subsequent IOL exchange using automated refractometry (RK-F2, Canon, Tokyo, Japan) and confirmed subjectively by in-house staff optometrists, using a 4 m lane and a -0.25 D adjustment, at least 3 months after surgery ^36^.

For the analysis, the predicted refraction was calculated using the optimized constants respective to each single constant formula included (Barrett A constant: 119.18, EVO 2.0 A constant 119.15, Haigis a0: 1.523, Hoffer Q pACD: 5.71, Hoffer QST pACD: 5.664, Holladay 1 SF: 1.859, Kane A constant: 119.16, PEARL-DGS A constant: 119.27, SRK/T A constant: 119.076). Refractive error was calculated by subtracting the predicted refraction from the actual postoperative refraction. The mean prediction error (ME), standard deviation (SD), root mean square error (RMSE) of the signed errors, median of absolute errors, and mean of absolute errors were calculated for the long-AL subgroup and the entire AL range. The Haigis formula was optimized separately for the triple constants (a0: 1.304/ a1: 0.442/ a2: 0.104). The CMAL was experimentally applied to the Haigis formula and optimized for the single (a0: 1.556) and triple constant (a0: 3.526/a1: 0.523/a2: 0).

To investigate the underlying causes of the optic-based newer formulas' myopic shifts observed in long Asian eyes, we conducted a comparison between the reference and predicted ELP of the single-optimized, CMAL-applied Haigis formula, which showed similar behavior and high correlations with the newer formulas (see Supplementary Fig. S1 and Table S1 online). This comparison was also performed for both single and triple-optimized versions of the formula for both conventional AL and CMAL applications to identify any potential differences between them. The reference ELP of the Haigis formula, which generates zero refractive error with both conventional AL and CMAL, was back-calculated^22^. The mean reference back-calculated and predicted ELP values were plotted against the conventional AL values by modifying the open-source code of the PEARL-DGS formula on GitHub^37^.

The association between the AL and ACD was plotted. The AL-ACD relationship of the training population for the PEARL-DGS formula was provided by the author. Among the 3100 eyes of 3100 patients, 133 had an AL > longer than 26 mm. To illustrate the relationship between ACD and the prediction error of each formula, the AL > 26 mm group was categorized into three subgroups by 1 standard deviation (SD) difference from the mean ACD: short (< 3.244 mm, 24 eyes), medium (3.244–3.777 mm, 86 eyes), and long (> 3.777 mm, 23 eyes). The ME for each subgroup within the AL > 26 mm category was then illustrated on a two-dimensional graph. Furthermore, for a comprehensive visualization across the entire AL range, all eyes were stratified into six AL subgroups (≤ 22 mm to > 26 mm). Each AL subgroup was divided into three additional subgroups according to ACD, based on a one SD interval from the mean ACD. The ME change in each formula was presented using a three-dimensional plot.

In addition, illustration of the ME according to AL changes using the older formulas (Hoffer Q^38^, Holladay 1^39^, and SRK/T^40^), which were not conducted in the previous study^18^, were performed to identify any potential difference from the previous reports^10–12,21,27,32^.

The normality of numerical errors was assessed using the Shapiro–Wilk test. Depending on whether the data is normally distributed, subsequent analysis were conducted using either the Student's t-test or the Wilcoxon signed-rank test to determine if the mean prediction error significantly differed from zero. For comparisons of the ME between formulas in the long eyes, to take into account both systematic and random error, the RMSE of each formula was compared using the heteroscedastic test^41^.

Statistical analyses were performed using Holladay’s software package^41^ based on R software (version 3.3.3; R Foundation, Vienna, Austria). A Holm-adjusted *P*-value < 0.05 was considered statistically significant.

### Supplementary Information

Supplementary Information.

## Data Availability

The datasets generated and/or analyzed during the current study are not publicly available but are available from the corresponding author upon reasonable request.
