# Supplementary material for: Investigation of the myopic outcomes of the newer intraocular lens power calculation formulas in Korean patients with long eyes
Source: Sci Rep. 2024 May 31;14:12558. doi: 10.1038/s41598-024-63334-y (PMC11143184; doi:10.1038/s41598-024-63334-y)
Supplement: Supplementary file 1 — Supplementary Information. [file 41598_2024_63334_MOESM1_ESM.docx]

**Supplementary Information**

**Investigation of the Myopic Outcomes of the Newer Intraocular Lens Power Calculation Formulas in Korean Patients with Long Eyes**

Jinchul Kim, Joonsung Park, Yoonjung Jo

Supplementary Table S1. Coefficients of determination (R^2^ values) between the prediction error of each formula (data from Reference 18)

| Formula | HSAL | HSCL |
| --- | --- | --- |
| PEARL | 0.6894 | 0.8503 |
| Barrett | 0.7027 | 0.7638 |
| EVO 2.0 | 0.7095 | 0.8076 |
| Kane | 0.7159 | 0.8157 |
| Hoffer QST | 0.8263 | 0.8272 |
| PEARL-rCMAL | 0.8429 | 0.81 |

| Formula | PEARL | PEARL-rCMAL |
| --- | --- | --- |
| Barrett | 0.8527 | 0.8501 |
| EVO 2.0 | 0.9503 | 0.9106 |
| Kane | 0.9435 | 0.9031 |
| Hoffer QST | 0.7582 | 0.8152 |

AL, axial length; CMAL, Cooke-modified axial length; HSAL, Haigis formula, single-optimized and AL-applied; HSCL, Haigis formula, single-optimized and CMAL-applied; PEARL-rCMAL = PEARL-DGS formula with reversed CMAL (= ([AL + 0.05467 × lens thickness – 1.28353] / 0.95855); R, Pearson correlation coefficient; EVO, Emmetropia Verifying Optical formula; Hoffer QST, Hoffer Q/Savini/Taroni formula; PEARL–DGS, Prediction Enhanced by Artificial Intelligence and output Linearization–Debellemanière, Gatinel, and Saad.

Supplementary Table S2. Refractive results of the patient population (data from Reference 18)

1. Refractive Results of the whole group (n = 3,100)

| Formula | ME | SD | MedAE | MAE |
| --- | --- | --- | --- | --- |
| PEARL | -2.0E-06 | 0.3175 | 0.2040 | 0.2481 |
| HTCL | -0.0015 | 0.3133 | 0.2011 | 0.2457 |
| Kane | 0.0001 | 0.3168 | 0.2031 | 0.2480 |
| EVO 2.0 | -1.2E-05 | 0.3171 | 0.2042 | 0.2483 |
| HTAL | -0.0019 | 0.3221 | 0.2133 | 0.2532 |
| HSAL | 0.0006 | 0.3226 | 0.2115 | 0.2542 |
| HSCL | 2.4E-05 | 0.3260 | 0.2109 | 0.2561 |
| Barrett | 0.0002 | 0.3282 | 0.2125 | 0.2577 |
| Hoffer QST | -0.0001 | 0.3358 | 0.2230 | 0.2660 |
| Holladay 1 | -3.4E-06 | 0.3505 | 0.2360 | 0.2782 |
| Hoffer Q | -5.8E-06 | 0.3534 | 0.2375 | 0.2805 |
| SRK/T | -0.0004 | 0.3902 | 0.2556 | 0.3091 |

**b)** Refractive results in the long eyes (>26 mm, n = 133)

| Formula | ME | P-value | SD | RMSE | MedAE | MAE |
| --- | --- | --- | --- | --- | --- | --- |
| PEARL | -0.2933 | **1.3E-15*** | 0.3410 | 0.4471 | 0.2914 | 0.3581 |
| HTCL | -0.0585 | 0.4668 | 0.3344 | 0.3383 | 0.2158 | 0.2608 |
| Kane | -0.2245 | **5.6E-11*** | 0.3347 | 0.4019 | 0.2669 | 0.3212 |
| EVO 2.0 | -0.2707 | **6.1E-14*** | 0.3409 | 0.4343 | 0.2958 | 0.3484 |
| HTAL | -0.0273 | 0.0731 | 0.3385 | 0.3383 | 0.2061 | 0.2585 |
| HSAL | -0.0062 | 0.9356 | 0.3400 | 0.3388 | 0.1991 | 0.2586 |
| HSCL | -0.2323 | **2.9E-11*** | 0.3433 | 0.4135 | 0.2652 | 0.3274 |
| Barrett | -0.2420 | **1.3E-13*** | 0.3377 | 0.4144 | 0.2738 | 0.3327 |
| Hoffer QST | -0.2702 | **9.5E-14*** | 0.3513 | 0.4421 | 0.3130 | 0.3596 |
| Holladay 1 | 0.0987 | **0.0077*** | 0.3909 | 0.4018 | 0.2824 | 0.3271 |
| Hoffer Q | 0.1150 | **0.0005*** | 0.3913 | 0.4065 | 0.2490 | 0.3199 |
| SRK/T | -0.1222 | **0.0010*** | 0.3923 | 0.4095 | 0.3033 | 0.3372 |

AL, axial length; CMAL, Cooke-modified axial length; HSAL, Haigis formula, single-optimized and AL-applied; HSCL, Haigis formula, single-optimized and CMAL-applied; HTAL, Haigis formula, triple-optimized and AL-applied; HTCL, Haigis formula, triple-optimized and CMAL-applied; MAE, mean absolute error; ME, mean numerical prediction error; MedAE, median absolute error; RMSE, root mean square numerical error; SD, standard deviation; EVO, Emmetropia Verifying Optical formula; Hoffer QST, Hoffer Q/Savini/Taroni formula; MAE, mean absolute error; ME, mean numerical prediction error; MedAE, median absolute error; PEARL–DGS, Prediction Enhanced by Artificial Intelligence and output Linearization–Debellemanière, Gatinel, and Saad; RMSE, root mean square numerical error; SD, standard deviation.

The optimized constants for the formulas are: Hoffer Q pACD: 5.71, Holladay 1 SF: 1.859, SRK/T A constant: 119.076. For the Haigis formula, HSAL a0: 0.523, a1: 0.4, a2: 0.1, HTAL a0: 1.304, a1: 0.442, a2: 0.104, HSCL a0: 1.556, a1: 0.4, a2: 0.1, HTCL a0: 3.526, a1: 0.523, a2: 0, PEARL, A constant: 119.27; Kane, A constant: 119.16; EVO 2.0, A constant: 119.15; Barrett, A constant: 119.18; and Hoffer QST, pACD: 5.664.

The bolded numbers with asterisks (*) in P-value column represent significant differences of

the ME of each formula from 0.

Supplementary Table S3. Statistical comparison of the root mean square error of the formulas for the long axial length subgroup with adjusted *P*-values (heteroscedastic test and Holm correction) (data from Reference 18)

| Formula | HTCL | Kane | EVO 2.0 | PEARL | HTAL | HSAL | HSCL | Barrett |
| --- | --- | --- | --- | --- | --- | --- | --- | --- |
| HTCL | - | - | - | - | - | - | - | - |
| Kane | **0.0E+00*** | - | - | - | - | - | - | - |
| EVO 2.0 | **0.0E+00*** | **0.0E+00*** | - | - | - | - | - | - |
| PEARL | **0.0E+00*** | **0.0100*** | 0.0811 | - | - | - | - | - |
| HTAL | 0.9530 | **0.0E+00*** | **0.0E+00*** | **0.0E+00*** | - | - | - | - |
| HSAL | 0.9530 | **0.0100*** | **0.0E+00*** | **0.0E+00*** | 0.8411 | - | - | - |
| HSCL | **0.0E+00*** | 0.7078 | **0.0E+00*** | **0.0E+00*** | **0.0E+00*** | **0.0100*** | - | - |
| Barrett | **0.0E+00*** | 0.4134 | **0.0100*** | **0.0100*** | **0.0E+00*** | **0.0100*** | 0.9530 | - |
| Hoffer QST | **0.0E+00*** | **0.0E+00*** | 0.9530 | 0.9530 | **0.0E+00*** | **0.0E+00*** | **0.0E+00*** | **0.0E+00*** |

The bolded values with asterisks (*) represent significant differences between the formulas compared.

AL, axial length; CMAL, Cooke-modified axial length; HSAL, Haigis formula, single-optimized and AL-applied; HSCL, Haigis formula, single-optimized and CMAL-applied; HTAL, Haigis formula, triple-optimized and AL-applied; HTCL, Haigis formula, triple-optimized and CMAL-applied; EVO, Emmetropia Verifying Optical formula; Hoffer QST, Hoffer Q/Savini/Taroni formula; PEARL–DGS, Prediction Enhanced by Artificial Intelligence and output Linearization–Debellemanière, Gatinel, and Saad.


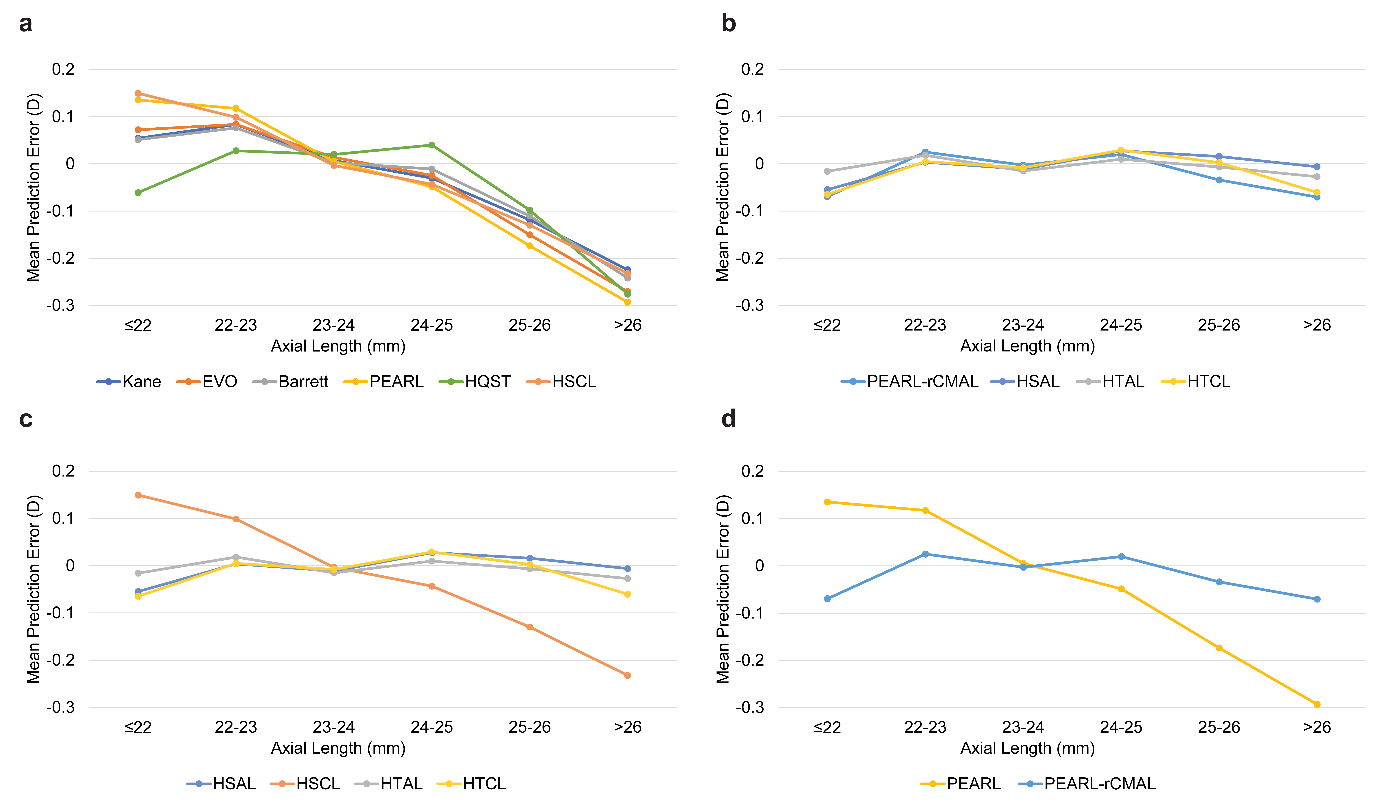


**Supplementary Fig S1****. Mean prediction error changes of each formula according to axial length (AL)** (figure from Reference 18)

1. Optimized ewer formulas and single-optimized, Cooke-modified AL (CMAL)-applied Haigis formula.
2. Haigis formulas and reversed CMAL-applied prediction enhanced by artificial intelligence and output linearization–Debellemanière, Gatinel, and Saad (PEARL–DGS) formula.
3. Haigis formula single-optimized, AL-applied; single-optimized, CMAL-applied; triple-optimized, AL-applied; and triple-optimized, CMAL-applied.
4. PEARL–DGS formulas: conventional AL-applied, and reversed CMAL-applied

AL: axial length; CMAL: Cooke-modified axial length; EVO: Emmetropia Verifying Optical formula; HSAL: Haigis formula, single-optimized and AL-applied; Hoffer QST: Hoffer Q/Savini/Taroni formula; HSCL: Haigis formula, single-optimized and CMAL-applied; HTAL: Haigis formula, triple-optimized and AL-applied; HTCL: Haigis formula, triple-optimized and CMAL-applied; PEARL–DGS: Prediction Enhanced by Artificial Intelligence and output Linearization–Debellemanière, Gatinel, and Saad; PEARL-rCMAL: PEARL–DGS formula with reversed CMAL (AL + 0.05467 × lens thickness [LT] – 1.23853) / 0.95855)-applied.


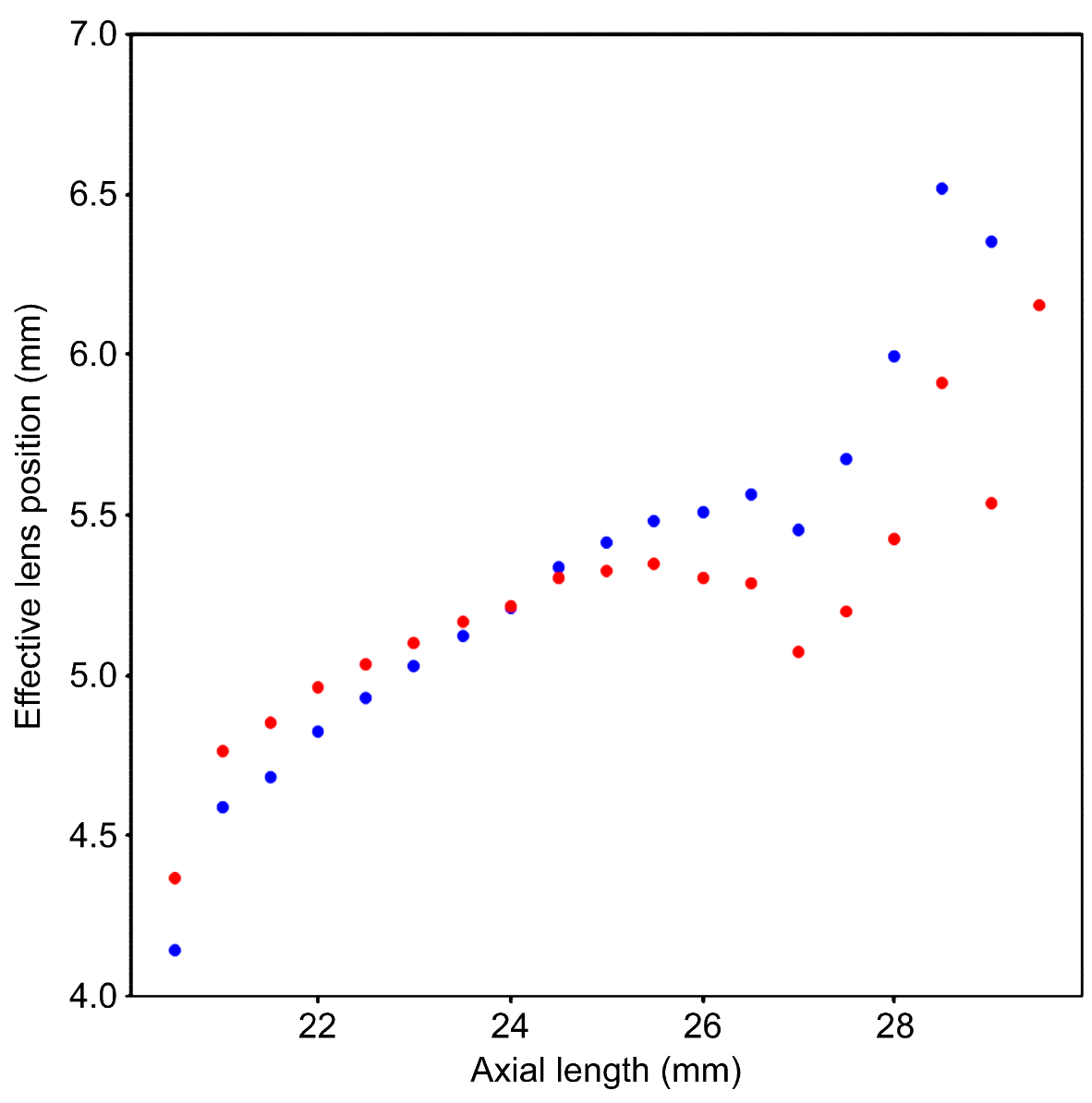


**Supplementary Fig S2. Back-calculated effective lens position (ELP) of conventional AL-applied Haigis formula (blue dots) versus back-calculated ELP of CMAL-applied Haigis formula (red dots).** The mean ELP values are plotted against conventional AL values rounded to 0.5 mm.

**
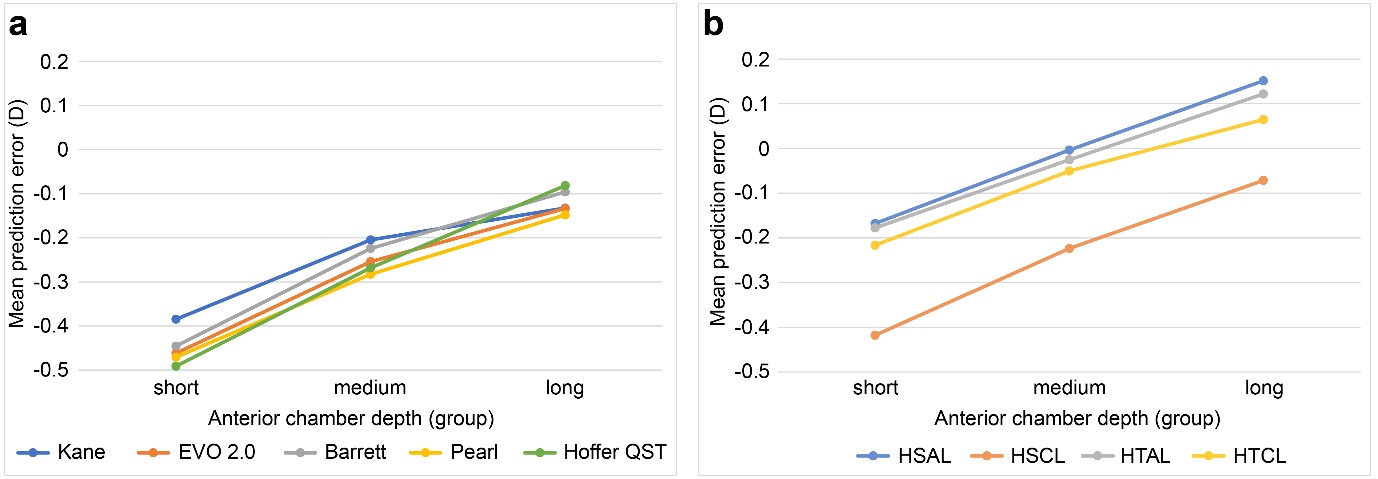
**

**Supplementary Fig S3.** Mean prediction error changes of each formula according to the ACD in the eyes with an AL > 26 mm (n=133). **a**) Newer formulas using their respective optimized constants. **b**) Haigis formula single-optimized, AL-applied; single-optimized, CMAL-applied; triple-optimized, AL-applied; and triple-optimized, CMAL-applied.

short = ACD < 3.244 mm (n = 24); medium = 3.244 mm < ACD < 3.777 mm (n = 86); long = ACD > 3.777 mm (n = 23)

ACD = anterior chamber depth; AL = axial length; CMAL = Cooke-modified axial length; EVO = Emmetropia Verifying Optical formula; Hoffer QST: Hoffer Q/Savini/Taroni formula; HSAL = Haigis formula, single-optimized and AL-applied; HSCL = Haigis formula, single-optimized and CMAL-applied; HTAL = Haigis formula, triple-optimized and AL-applied; HTCL = Haigis formula, triple-optimized and CMAL-applied; Pearl = Prediction Enhanced by Artificial Intelligence and output Linearization – Debellemanière, Gatinel, and Saad.


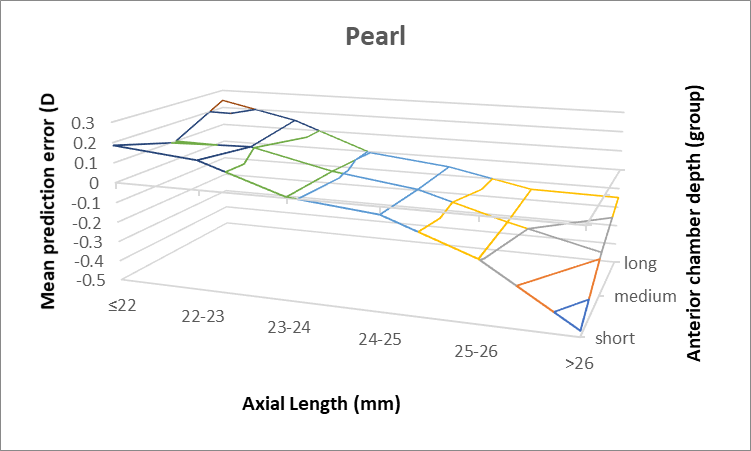


**Supplementary Fig S4.** Mean prediction error changes of each formula according to the ACD in the entire AL range (n=3,100). Stratification of each AL subgroup into three ACD categories based on one standard deviation from the mean ACD

ACD = anterior chamber depth; AL = axial length; CMAL = Cooke-modified axial length; EVO = Emmetropia Verifying Optical formula; Hoffer QST: Hoffer Q/Savini/Taroni formula; HSAL = Haigis formula, single-optimized and AL-applied; HSCL = Haigis formula, single-optimized and CMAL-applied; HTAL = Haigis formula, triple-optimized and AL-applied; HTCL = Haigis formula, triple-optimized and CMAL-applied; Pearl = Prediction Enhanced by Artificial Intelligence and output Linearization – Debellemanière, Gatinel, and Saad.


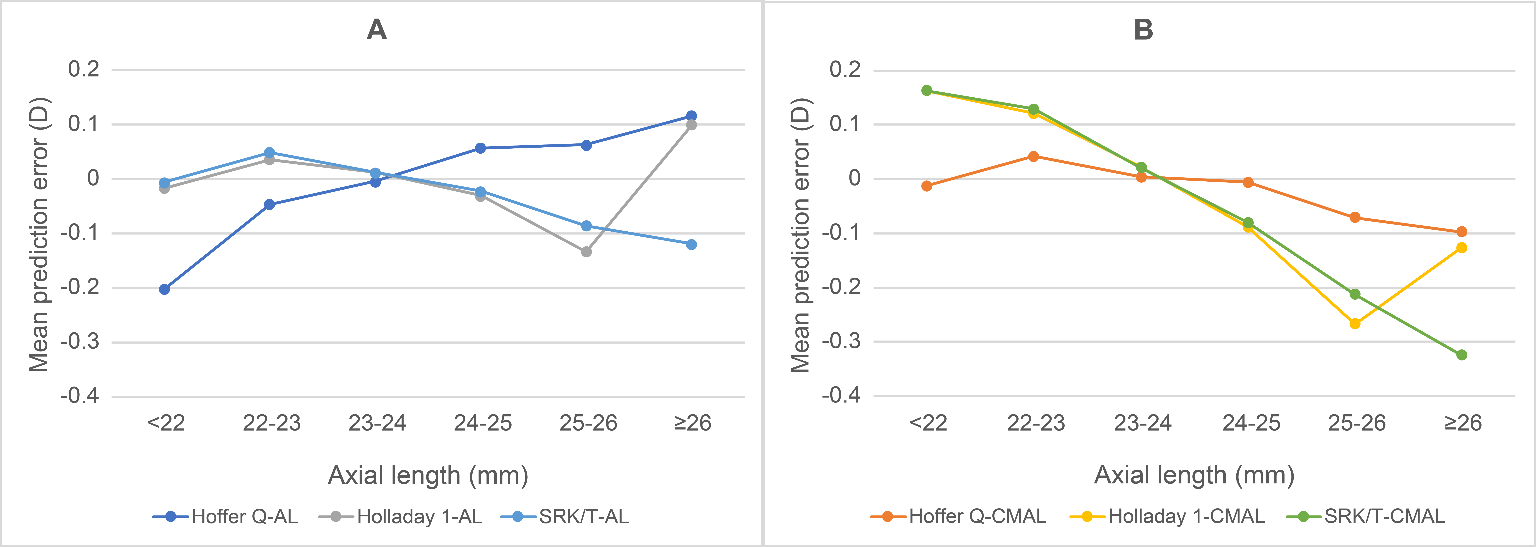


**Supplementary Fig S5**. **Mean prediction error changes of each older formula according to axial length (AL).**

**a**) Formulas with conventional AL application.

**b**) Formulas with CMAL application.

AL: axial length; CMAL: Cooke-modified axial length


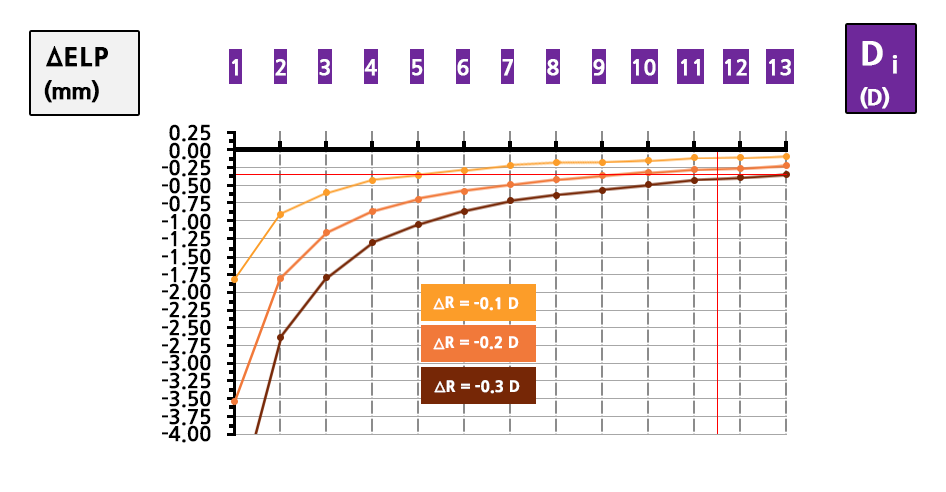


**Supplementary Fig S6. Theoretical simulation of ELP difference (ΔELP) of -0.35 mm to induce a specific variation in refraction (ΔR) with an IOL power (Di) of 11.5 D and a corneal power of 43 D** (figure derived from Reference 24, and modified to enhance visual clarity)

The intersection of red horizontal and vertical lines corresponds to approximately -0.25 D refraction change.

D: Diopters; ELP: Effective lens position; IOL: intraocular lens


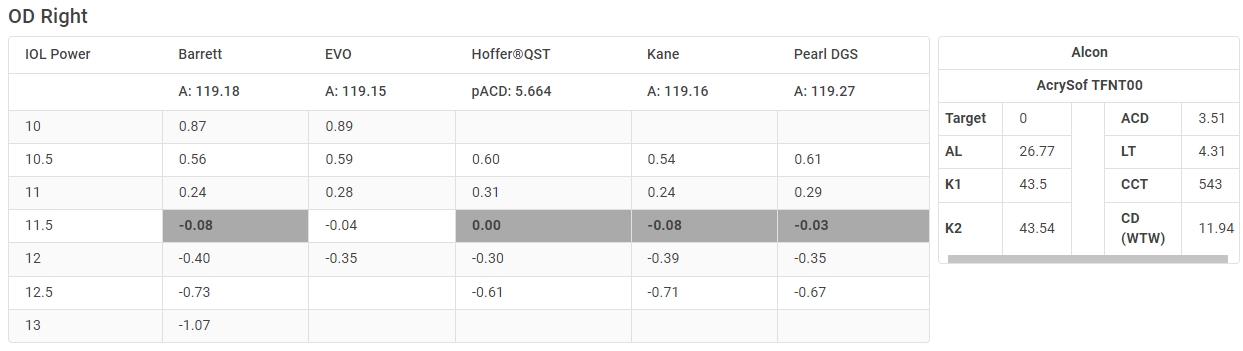


**Supplementary Fig S7. The postoperative refraction prediction results and assigned intraocular lens (IOL) powers of the newer IOL calculation formulas** (figure from the European Society of Cataract and Refractive Surgeons online calculator: <https://iolcalculator.escrs.org/>, accessed December 5, 2023)

A: A constant; ACD: anterior chamber depth; AL: axial length; CCT: central corneal thickness; CD: corneal diameter; EVO: Emmetropia Verifying Optical formula; Hoffer QST: Hoffer Q/Savini/Taroni formula; IOL: intraocular lens; K1: keratometry of flat meridian; K2: keratometry of steep meridian; LT: lens thickness; pACD: personalized ACD. PEARL–DGS: Prediction Enhanced by Artificial Intelligence and output Linearization–Debellemanière, Gatinel, and Saad; WTW: white to white
